# Correction to: Myeloid-derived suppressor cells cross-talk with B10 cells by BAFF/BAFF-R pathway to promote immunosuppression in cervical cancer

**DOI:** 10.1007/s00262-022-03304-3

**Published:** 2022-10-25

**Authors:** Ding Jianyi, Gan Haili, Yin Bo, Yang Meiqin, Huang Baoyou, Hu Haoran, Li Fang, Zheng Qingliang, Han Lingfei

**Affiliations:** 1 Department of Gynecology, Shanghai First Maternity and Infant Hospital, Tongji University School of Medicine, Shanghai, 201240 China; 2 Department of Gynecology, School of Medicine, Shanghai East Hospital, Tongji University, Shanghai, 200120 China; 3 Sun Yat-sen University, Prenatal Diagnosis Center, The Eighth Affiliated Hospital, Shenzhen, 518000 People’s Republic of China

## Correction to: Cancer Immunology, Immunotherapy https://doi.org/10.1007/s00262-022-03226-0

The original version of this article unfortunately contained a mistake. The statistical chart in Fig. 1c, d as the same as that in Fig. 1a, b.

**Fig. 1:**
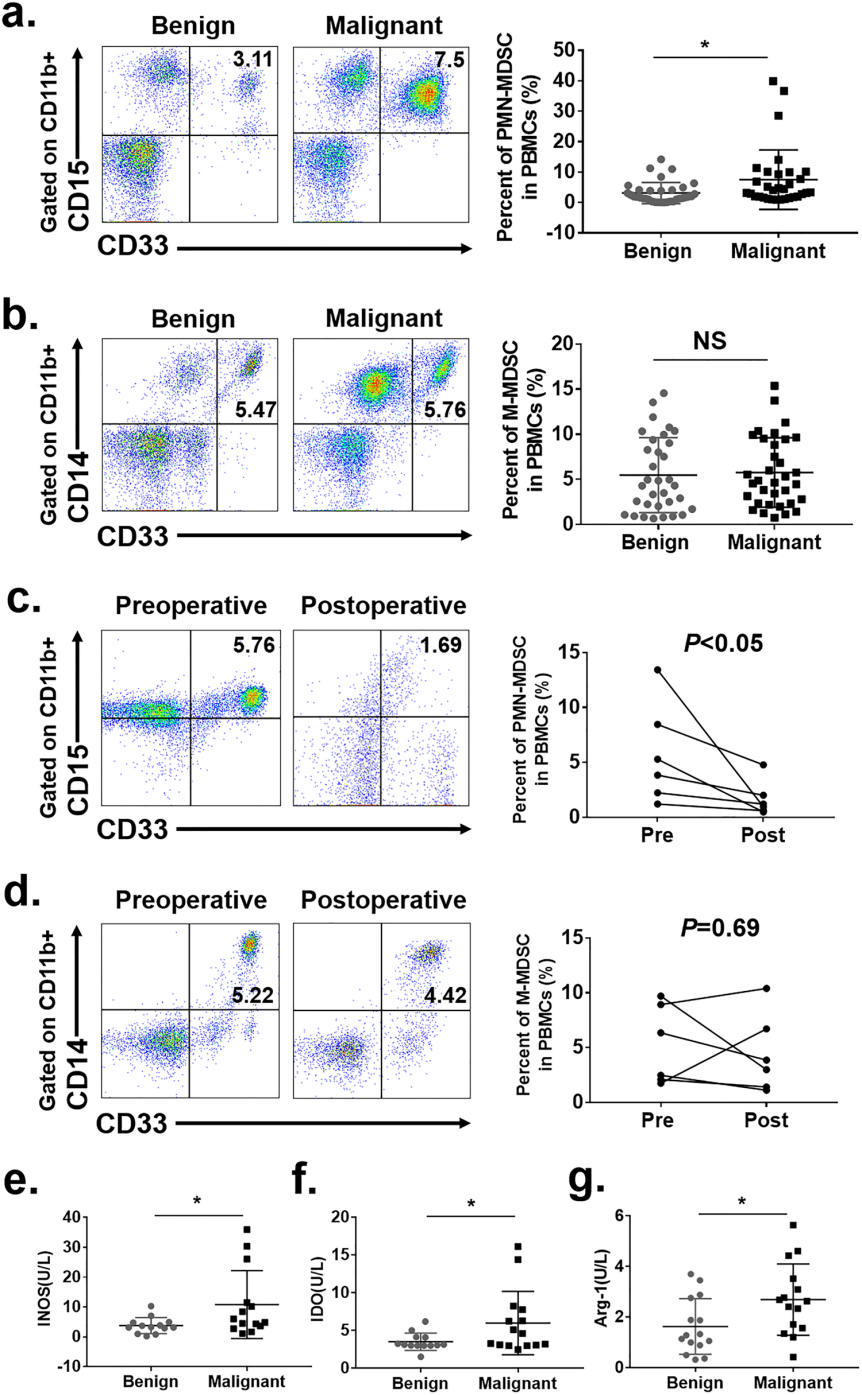
Accumulation of MDSCs in cervical cancer patients. **a** Flow cytometry analysis of PMN-MDSCs in peripheral blood from patients with benign or malignant tumors. Statistical analysis of the frequencies of PMN-MDSCs as a percentage of the mononuclear cells (MCs) population. **b** Flow cytometry analysis of M-MDSCs in peripheral blood from patients with benign or malignant tumors. Statistical analysis of the frequencies of M-MDSCs as a percentage of the MCs population. **c** Flow cytometry analysis of the frequency of PMN-MDSCs in the peripheral blood of patients with cervical cancer before and 5 days after surgery. Statistical analysis of the frequencies of PMN-MDSCs as a percentage of the MCs population. **d** Flow cytometry analysis of the frequency of M-MDSCs in the peripheral blood of patients with cervical cancer before and 5 days after surgery. Statistical analysis of the frequencies of M-MDSCs as a percentage of the MCs population. **e**–**g** ELISA analysis of the concentrations of IDO, INOS and Arg-1 in the peripheral blood of patients with either malignant or benign cervical tumors. Each point corresponds to an individual patient. Lines indicate the 25th to 75th percentiles. Horizontal lines represent the median value. Data were analyzed using Student’s t test and are expressed as the mean ± SD. Symbols represent statistical significance (*p < 0.05)

The corrected Fig. 1 is given in the following page.

